# Synergistic Effects of Flower Color and Mechanical Barriers on Pollinator Selection Within the Papilionoideae of Fabaceae

**DOI:** 10.3390/plants14111568

**Published:** 2025-05-22

**Authors:** Xiang Zhao, Ruochun Gao, Jie Bai, Jing Rong, Xuexia Wei, Hairong Wang, Xiaojuan Zhu, Kun Sun, Qinzheng Hou

**Affiliations:** College of Life Sciences, Northwest Normal University, Lanzhou 730070, China; xiangzhao@nwnu.edu.cn (X.Z.); 18215143109@163.com (R.G.); 18298807303@163.com (J.B.); 17318781261@163.com (J.R.); 18293016742@163.com (X.W.); 17789365752@163.com (H.W.); 15117244369@163.com (X.Z.)

**Keywords:** Papilionoideae, mechanical screening mechanisms, flower color, cooperative evolution

## Abstract

Current understanding of synergistic trait effects in plant–pollinator systems remains limited, particularly regarding combined visual and mechanical screening mechanisms. Given the specialized flower opening mechanisms and diverse color signals in the Papilionoideae of Fabaceae, this study examines how floral color and mechanical traits jointly mediate pollinator selection in five co-flowering sympatric species. The flower structure of Papilionoideae typically features a keel formed by fused petal lobes that encloses reproductive organs, with flower operative strength thresholds directly reflecting the mechanical resistance required to dehisce the keel and access nectar/pollen. Flower operative strength thresholds and insect mechanical capabilities were quantified, and visitation behaviors were observed under natural conditions. Significant interspecific variation in flower mechanical strength (12.59–20.25 mN) was identified, with visiting insects consistently exhibiting strengths exceeding these thresholds, suggesting mechanical barriers selectively filter pollinators. Non-visiting insects exhibited either insufficient or excessive strength relative to floral thresholds, which is related to the flower-visiting preferences of different insects. Although no linear correlation was found between flower color (RGB color space) and mechanical strength, the combined analysis revealed synergistic screening where color attracted specific pollinators from a subset capable of overcoming mechanical barriers. These findings demonstrate that flower color and mechanical traits function as complementary filters, optimizing pollinator efficiency and excluding ineffective visitors. The study highlights the necessity to explore multi-trait interactions in plant–pollinator co-evolution, with implications for biodiversity conservation and ecosystem service management.

## 1. Introduction

The interactions between plants and their pollinators are pivotal in shaping ecosystem diversity and ensuring the continuation of essential ecosystem services [1]. Floral traits are widely acknowledged to play a central role in shaping pollinator communities and driving the evolution of plant–pollinator interactions. These traits act as selective filters, ensuring that only specific pollinators can effectively access floral rewards and transfer pollen [2]. This view is strongly supported by the concept of pollination syndromes, which posits that floral traits evolve to attract specific groups of pollinators. Among the numerous strategies flowering plants employ to filter suitable pollinators, two prominent mechanisms are flower color, which acts as a visual signal, and mechanical screening, which serves as a physical threshold [3,4].

Flower color has evolved as a visual attractant that conveys specific signals to potential pollinators [5]. It has been demonstrated that different pollinators exhibit distinct color preferences, which are often aligned with their visual systems [6]. For instance, bumblebees are typically attracted to blue and purple hues. In contrast, honeybees and hoverflies often prefer yellow and white flowers [7], while butterflies are usually attracted to red and yellow flowers [8]. These color preferences are generally shaped by the pollinator’s ability to detect and differentiate specific wavelengths and their association with floral rewards [9]. Lunau et al. [10] demonstrated that flower color serves as a guide that helps pollinators efficiently locate flowers and forage for nectar and pollen. This visual cue increases the likelihood that flowers attract effective pollinators and dissuades those that might be less capable of providing pollination services [11]. In environments characterized by high biodiversity and competitive floral displays, color differentiation can enhance the specificity of plant–pollinator interactions, improving pollination efficiency [12]. However, color-based attraction alone cannot explain the high specificity observed in many plant–pollinator systems.

On the other hand, mechanical screening mechanisms act as a barrier that selectively allows only pollinators with specific physical capabilities to access floral rewards [13]. Mechanical screening mechanisms, such as corolla length, floral constriction, and the presence of movable floral parts, act as physical barriers to filter pollinators based on their morphology or strength [14]. These structures are proposed to exclude pollinators lacking the required body size, strength, or feeding techniques. For example, *Phlomis fruticosa* L. belongs to the family Lamiaceae, and it has been shown that the closed corollas of *Phlomis fruticosa* act as mechanical devices to select effective pollinators [15]. Similarly, flowers of the *Delphinium caeruleum* Jacq. ex Camb. (Ranunculaceae) require downward pressure on the staminodes to release pollen, a mechanism that favors bumblebees with greater strength [13]. Also, *Pedicularis longiflora* Rudolph (Orobanchaceae) has a long, curved corolla tube, a unique floral structure that makes its pollination mechanism more specialized [16]. The longer corolla tube allows only pollinators with appropriately sized mouthparts (e.g., specific species of bees) to effectively contact the pollen. This specialized pollination mechanism helps increase pollination efficiency, reduce pollen wastage, and ensure that pollen is delivered to the right receptor [17]. These examples demonstrate how mechanical traits such as tube length or movable structures act as filters to exclude unsuitable pollinators. Although color and mechanical traits are often studied independently, emerging evidence suggests they may function interdependently. For example, Gurevich et al. [18] demonstrated that floral color patterns could guide pollinators in interacting with morphologically complex structures. For instance, long corolla tubes in hummingbird-pollinated flowers are often coupled with red coloration to filter out inefficient visitors, demonstrating how traits act in concert to mediate pollinator specificity [19]. Similarly, Wang et al. [20] proposed that visual and physical traits could act synergistically to reinforce pollinator specificity. However, empirical evidence for this hypothesis remains limited, especially in plant populations with different flower colors.

**Figure 1:**
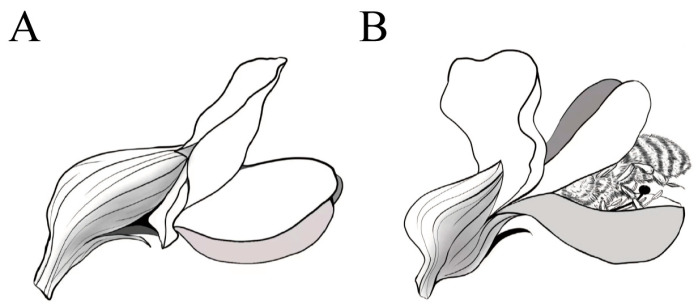
Schematic diagram of plants in the subfamily Papilionoideae. (**A**) Schematic representation of the floral parts. (**B**) Schematic diagram of insects visiting flowers.

Given that flower color and mechanical barriers play critical roles in attracting and filtering pollinators, the interplay between these factors could lead to important insights into plant–pollinator co-evolution [21]. The paucity of research on the combined effect of these two mechanisms presents a unique opportunity to investigate their interactions and impacts. Papilionoideae plants were selected as the subjects of study due to their distinctive floral opening mechanism and variety of colors, as they tend to use a heterogamous pollination mechanism, relying on pollinators such as insects [22]. First, Papilionoideae species exhibit remarkable diversity in flower color, ranging from white and yellow to purple and red, which may target different pollinator groups. Second, the papilionaceous corolla—composed of a banner, wings, and keel—creates a specialized mechanical system (Figure 1). The keel petals, which enclose reproductive organs, require pollinators to exert pressure to trigger pollen release, effectively filtering out visitors lacking sufficient strength or behavioral adaptations. The flowers in Papilionoideae plants are typical vexillary flowers, with hidden nectar and pollen. For instance, the vexillum mainly serves as an advertisement, and most of the nectar channels are located at the base of the vexillum. The keel petal provides a landing platform for bees, allowing insects to alight and delve deep into the flower. Nectar is the plant’s reward for pollinators’ visits and one of the main reasons pollinators visit flowers [23]. Papilionoideae species are ecologically and agriculturally significant, making them a priority for understanding pollination strategies [24]. This combination of visual and mechanical complexity provides an ideal framework to investigate how dual filtering mechanisms operate in tandem to shape pollinator assemblages. Thus, the present study aims to explore the synergistic effects of flower color and mechanical screening mechanisms on the selection of pollinators in Papilionoideae plants. Specifically, it addresses (1) how Papilionoideae flower color and mechanical strength act as complementary filters for pollinator species and (2) whether different pollinators exhibit preferences for specific combinations of Papilionoideae flower colors and mechanical barriers.

## 2. Results

### 2.1. Correlations Among Floral Traits and Biomechanics

**Figure 2:**
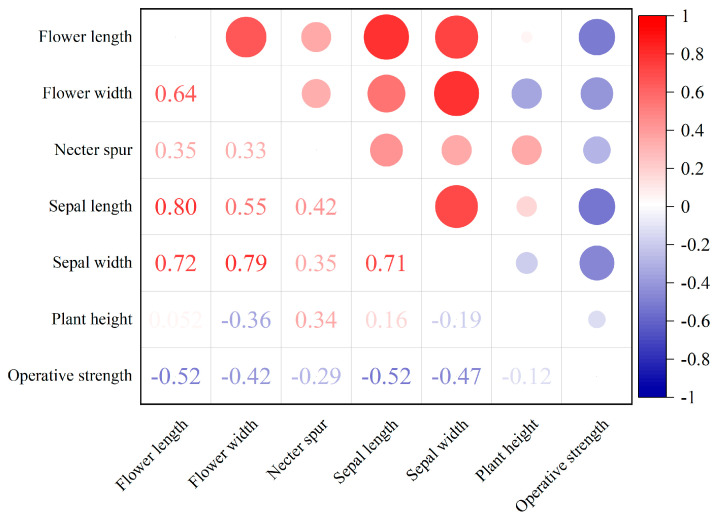
Heatmap illustrating the correlation coefficients among various plant morphological traits, including flower length, flower width, nectar spur length, sepal length, sepal width, plant height, and operative strength. The color gradient ranges from blue (indicating negative correlations) to red (indicating positive correlations), with the correlation coefficients displayed within each cell. Different circle sizes represent the magnitude of correlation coefficients (larger circles indicate stronger correlations). The scale bar on the right represents the strength of the correlation, from −1 to 1 (*p* < 0.05).

The analysis revealed significant variation in floral traits among the species (Table 1). *V. bungei* exhibited the highest operative strength (20.25 ± 2.88 mN) and the largest flower dimensions. At the same time, *S. flavescens* and *O. ochrantha* var. *longisepala* demonstrated lower operative strengths (12.59 ± 2.23 mN and 13.23 ± 3.10 mN, respectively), consistent with their smaller flower and sepal sizes. Correlation analysis (Figure 2) revealed strong positive relationships between operative strength and several floral morphological traits. Plant height showed a moderate negative correlation with operative strength, suggesting taller plants may invest less in mechanical barriers.

### 2.2. Observation of Visiting Insects

**Figure 3:**
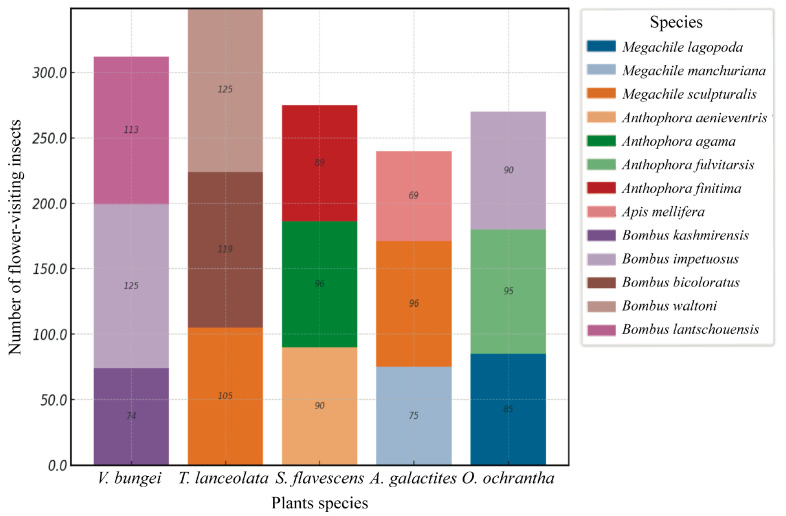
Stacked bar plot showing the relative abundance of different pollinator species visiting five Papilionoideae plants. Each colored segment within the bars represents a specific pollinator species, with numeric values indicating the actual count of visits. The legend on the right identifies the pollinator species corresponding to each color.

The study results indicated that there were 17 main flower visitors to the plants at the research site. Except for four insect species—*Megachile rotundata* F. (Hymenoptera, Megachilidae), *Eristalis tenax* L. (Diptera, Syrphidae), *Apis cerana* Fabricius (Hymenoptera, Apidae), and *Ichneumon* L. sp. (Hymenoptera, Ichneumonidae)—which did not visit Papilionoideae plants, the remaining 13 species served as primary pollinators (Table S1; Figure 3). All observed insect visitors displayed a consistent behavioral pattern during flower visitation. Upon approaching a flower, the insect first landed on the wing petals. Subsequently, it exerted downward pressure on the keel petals using its forelegs and body, a mechanical action necessitating sufficient body mass and strength. This action exposed the stamens and pistil, enabling the insect to insert its proboscis, often with its head, into the flower to access nectar as a reward. During this process, the insect’s abdomen or dorsal side inevitably made contact with the flower’s reproductive structures, thereby facilitating pollen transfer and successful pollination. This interaction highlights the importance of all the flower’s mechanical and morphological traits in filtering suitable pollinators. The observed behavior underscores the role of mechanical strength as a key trait in ensuring that only efficient pollinators, capable of overcoming the floral barrier, contribute to reproductive success.

### 2.3. Strength of Pollinators

**Figure 4:**
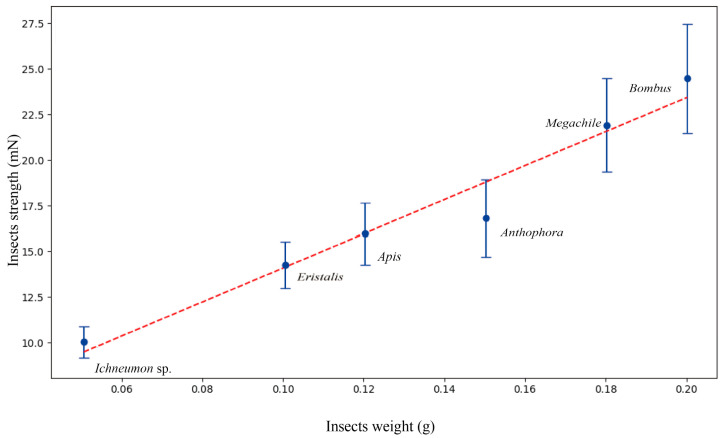
The scatter plot represents the observed data points for different insect genera, with genera on the *x*-axis ordered by decreasing weight (g) and strength (mN) on the *y*-axis. Error bars show the standard deviation (SD). The red dashed line indicates the linear regression line fitted to the data points.

Insect weight positively correlated with insect mechanical strength (Figure 4). As insect weight increased, mechanical strength also exhibited a tendency to increase. *Bombus* L. sp. (Hymenoptera, Apidae) exhibited the highest weight (approximately 0.20 g) and strength (27.56 mN). *Eristalis* L. sp. (Diptera, Syrphidae) and *Ichneumon* sp. exhibited both low weight and mechanical strength. On average, a honeybee can exert a force equivalent to 15 times its body weight.

### 2.4. Relationship Between Flower Operative Strength and Bee Strength

**Figure 5:**
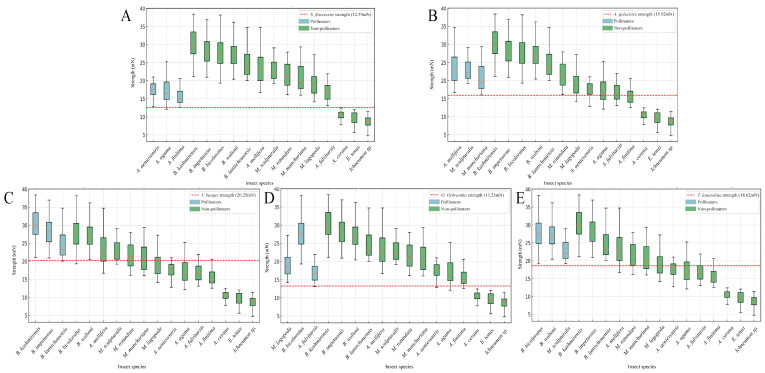
Mechanical strength of keeled petals of five Papilionoideae plants and insects pressed apart. Boxplots showing the strength distribution of various insect species, categorized into pollinators and non-pollinators. In the boxplots, the central line represents the mean, the box represents the interquartile range, and the whiskers represent the minimum–maximum values. In each plot, the blue boxes represent pollinators of this plants, while the green boxes represent non-pollinators of this plants. The red dashed lines mark the strength thresholds for each Papilionoideae species. (**A**) *S. flavescens*. (**B**) *A. galactites*. (**C**) *V. bungei*. (**D**) *O. ochrantha* var. *longisepala*. (**E**) *T. lanceolata*.

The relationship between flower operative strength and the mechanical strength of flower-visiting insects was analyzed across five Papilionoideae species. The results, presented in Figure 5A–E, illustrate the operative strength thresholds of each plant species, represented by the red dashed lines in each panel, along with the corresponding strength distributions of flower-visiting and non-flower-visiting insects. Each plant species exhibited a unique flower operative strength threshold, ranging from 12.59 mN (*S. flavescens*) to 20.25 mN (*V. bungei*). For all five Papilionoideae species, the mechanical strength of flower-visiting insects consistently exceeded, and in some cases was several times greater than, the operative strength thresholds of the flowers they pollinated. This highlights the critical role of sufficient mechanical strength in enabling successful flower visitation and pollination. However, the mechanical strength of non-visiting insects was either higher or lower than that of open flowers in five Papilionoideae species. This phenomenon may be related to the flower-visiting preferences of insects.

Among the 17 insect species observed and captured at the study site, *Apis cerana*, *Eristalis tenax*, and *Ichneumon* sp., all of which exhibited mechanical strengths below the operative thresholds of the Papilionoideae flowers, were not observed visiting these plants. Instead, these insects were found visiting other open-type flowers in the vicinity, which lacked mechanical barriers. In addition, although *Megachile rotundata* surpasses the mechanical threshold of flowers of all Papilionoideae plants, it does not visit plants of this subfamily. It is worth noting that for most plants in the Papilionoideae in this study, many insects have the force to open specific species but do not visit them. This indicates that the operational strength of flowers, as a mechanical barrier, may be one of the screening mechanisms for pollinators, and other factors may play a synergistic role in the screening process.

### 2.5. Flower Visiting Preferences of Insects

**Figure 6:**
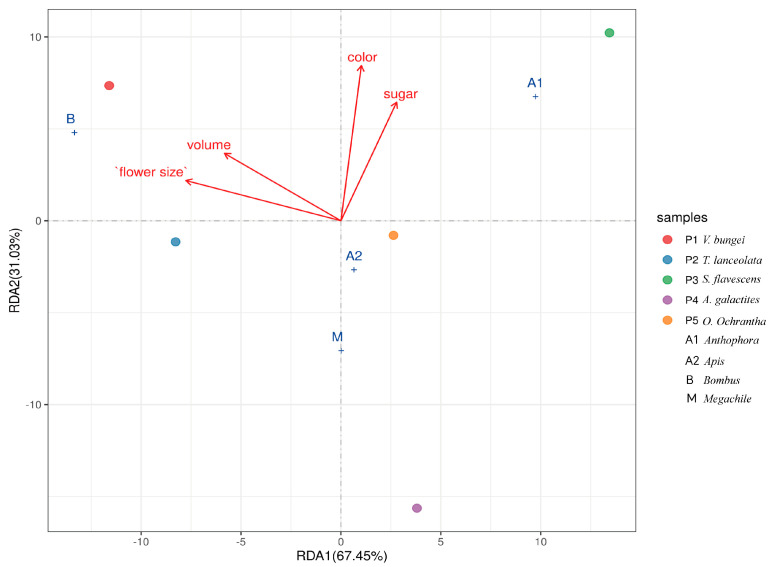
Redundancy analysis (RDA) plot illustrating the relationship between insect visits to five Papilionoideae species and floral traits (color, flower size, volume, sugar). Different-colored dots (P1–P5) represent five Papilionoideae species. Blue plus signs (A1, A2, M, B) represent different genera of flower-visiting insects. Red arrows indicate floral traits: color, sugar, volume, and flower size. RDA1 accounts for 67.45% of the variation, and RDA2 accounts for 31.03% of the variation, reflecting the main dimensions of the influence of floral traits on insect–plant interaction patterns. Detailed RDA results refer to Supplementary Materials (Tables S3 and S4).

From the Redundancy Analysis (RDA) plot (Figure 6), it can be visually observed that among the factors influencing insect visits to the five Papilionoideae, the arrows for “flower size” and “color” are relatively long. In an RDA plot, the length of an arrow typically represents the magnitude of a variable’s explanatory power. Combined with the data, “flower size” had a coefficient of −0.8988 along RDA1, and “color” exhibited a high coefficient of 0.9779 (Table S2) along RDA2. This result indicates that both “flower size” and “color” play a dominant role in attracting pollinators. This is because flower size is not only positively correlated with the force required to push open the keel petals of Papilionoideae plants, but also larger flowers are more conspicuous, thus affecting an insect’s ability to detect flowers.

**Figure 7:**
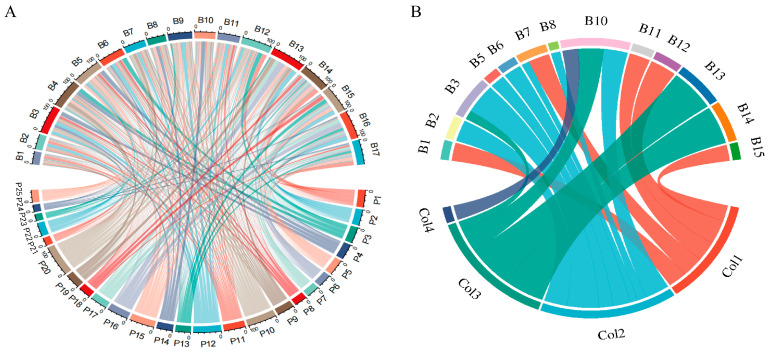
Illustrates the patterns of insect–plant interactions and flower-color preferences of insects. (**A**) Shows the interaction network between 17 species of flower-visiting insects (labeled as B1–B17) and 25 plant species (labeled as P1–P25). Each connection represents the visitation relationship, with the thickness and color of the lines indicating the strength and nature of the interactions. Insect and plant numbers are shown in Table S2. (**B**) Depicts the flower-color preferences of 13 insect species (labeled as B1–B3, B5–B8, B10–B15) for four flower-color categories (Col1: purple, Col2: white, Col3: yellow, Col4: blue). The connections from each insect species to the color categories visually represent their preferences, where thicker connections indicate a stronger preference for a particular flower color. The classification numbers of flower colors are shown in Table S5.

In Figure 7A, we observed a total of 25 plant species near the study site and identified 17 species of flower-visiting insects (B1–B17), as detailed in Table S5. The diagram reveals distinct differences in the visitation patterns of these insects to the 25 plants. In Figure 7B, which focuses on flower-color preferences, we narrowed our analysis to the 13 insect species that were observed visiting both the five Papilionoideae plants and non-Papilionoideae plants. The plants visited by these 13 insect species were classified into four flower-color categories (Col1: purple, Col2: white, Col3: yellow, Col4: blue; Table S5). Visually, each insect species demonstrated distinct preferences for different flower colors. Insect species such as B11, B12, and B15 were predominantly associated with the Col2 white-flower category, indicating a strong preference for this color. Their connections to Col2 in the diagram are notably thick, demonstrating a clear bias toward Col2 white flowers. This analysis highlights that color serves as an important factor in influencing the flower-visiting choices of different insect species.

### 2.6. Relationship Between Flower Color and Mechanistic Screening Mechanisms

**Figure 8:**
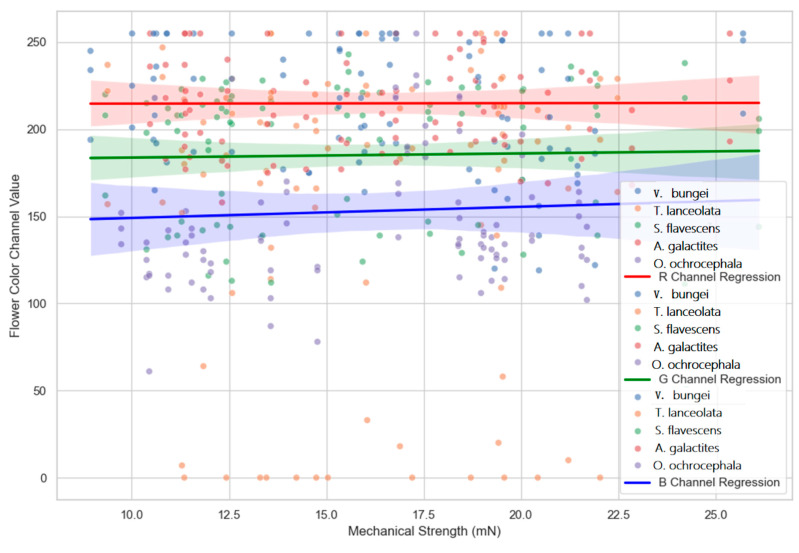
Relationship between mechanical strength (mN) and floral channel values for different plant species. Each point represents a sample, with different species indicated by color. The plot includes regression lines and shaded regions representing each species’ fit and confidence intervals.

The results (Figure 8) indicate no significant relationship between flower color values and the mechanical strength of flowers, with the *p*-value for the R channel being 0.0507, for the G channel 0.1483, and for the B channel 0.0888. This finding was consistent across all the analyzed species. While minor trends were observed in individual color channels (e.g., slight positive trends for the R and B channels), the overall relationship did not support a statistically significant correlation. These findings imply that flower color and mechanical screening mechanisms are not directly linked or dependent on one another as isolated factors.

## 3. Discussion

This study aimed to explore the synergistic relationship between flower color and mechanical strength in shaping plant–pollinator interactions, a topic that has been understudied. In comparison, previous studies have emphasized the independent roles of visual signaling and physical barriers in pollinator filtering. Our findings advance two key insights: (1) floral mechanical strength establishes a primary threshold for pollinator access, while flower color acts as a filter to refine pollinator preferences within mechanically competent visitors; (2) pollinator species exhibit distinct hierarchical decision-making processes, where mechanical capacity supersedes color preference, but color-driven selection emerges once mechanical thresholds are satisfied. This two-tiered selection framework reconciles conflicting perspectives in pollination ecology, where previous studies have emphasized either mechanical filtering or chromatic signaling as primary drivers of pollinator selection.

Larger flowers, which often possess greater mechanical strength, may have co-evolved with larger pollinators [25,26]. These flowers require a certain mechanical strength to withstand the physical pressure exerted by pollinators during visits, ensuring effective access to floral rewards (such as nectar) and efficient pollen transfer. Size-matching can act as a filter, excluding smaller, less effective pollinators that cannot navigate or withstand the mechanical properties of larger flowers [27]. This optimizes the plant’s resource allocation by focusing on pollinators that provide the most effective pollination service. Thus, there is also a connection between the size of the floral traits and the screening system. This is due to the fact that there is always a positive correlation between flower mechanical strength and flower size. Insects with insufficient mechanical ability did not visit, highlighting the importance of mechanical thresholds in legume pollination systems, and also supporting the “mechanical exclusion” hypothesis regarding the absolute limits imposed by floral structures on pollinator access [28].

Flower size, beyond influencing mechanical strength, is a key factor in attracting pollinators. Larger flowers, with their greater display area, are often preferred by pollinators [29,30,31,32]. Similarly, as a visual cue for attracting insects, color also has a significant impact on the type and behavior of pollinators [33]. Despite the correlation analysis results indicating a lack of significant direct correlation between flower color and mechanical strength, it is interesting to note that the RDA analysis reveals the dominant role of traits related to floral display (flower size and flower color) in attracting insects. This is not difficult to understand, as flower size and color are often the first signals received by insects when visiting flowers. Considering the dual impact of flower size on mechanical strength and pollinator attraction (affecting the display area of flower color), we suggest that there may be a dual screening of mechanical strength and color for pollinators in the Papilionoideae, which, to some extent, also challenges the assumption of independent trait evolution. We also found that *A. cerana*, *E. tenax*, and *Ichneumon* sp. lack the mechanical strength to open legume flowers and instead visit other flowers that are already open. Furthermore, many insects in this study can open specific Papilionoideae species but choose not to. This suggests that color may act as a complementary signal to reinforce the mechanical filter. For instance, *Bombus* species preferentially visited flowers with specific color spectra despite overcoming mechanical barriers [34], aligning with Koski ’s [35] model, where color enhances pollinator fidelity in mechanically compatible groups, reducing interspecific pollen transfer. The exceptions of *Megachile manchuriana* Yasumatsu (Hymenoptera, Megachilidae) and *Bombus lantschouensis* Vogt (Hymenoptera, Apidae) suggest additional olfactory or nectar chemistry cues [36], highlighting floral filtering’s multidimensional nature. However, our study extends this concept: even when insects possess adequate strength (e.g., *Bombus* sp.), their visitation patterns are modulated by color preferences—a finding consistent with Ruxton and Schaefer’s [33] assertion that color serves as a “post-filter” after mechanical compatibility is satisfied.

Our study proposes that flower color and mechanical strength act synergistically as a composite screening mechanism, aligning with Caruso et al. [37]’s suggestion of multiple-trait interaction to fine-tune pollinator interactions. This reinforces that complex plant–pollinator relationships depend on both individual traits and their interactions. The absence of a direct statistical relationship between color (RGB) and mechanical strength implies context-dependent interactions, challenging the traditional view of isolated flower traits and suggesting an integrated model of co-evolution. Moreover, our research shows that while flower color guides pollinator decisions, its effectiveness depends on prior mechanical compatibility—a point often overlooked in sensory ecology. This hierarchical interaction may explain why conspicuous flowers sometimes receive limited visitation [38], indicating mechanical filters constrain color attraction. Our results contribute to debates on evolutionary pressures: though some studies [39] suggest visual and physical traits enhance pollinator selectivity, the lack of a direct color-mechanical correlation implies a more nuanced interpretation. Other factors like scent or nectar composition, highlighted in studies [40] but not considered here, may interact with color and mechanical traits. Our findings align with Lagomarsino et al. [41], showing that flowers with strong mechanical barriers can attract pollinators capable physically and driven by color, optimizing pollination efficiency.

In conclusion, our study reveals how floral traits, especially color and mechanical strength, function synergistically to influence pollinator behavior. This multi-trait interaction is a sophisticated evolutionary strategy for selecting efficient pollinators. While the lack of a direct color-mechanical correlation suggests non-straightforward evolution, their combined effects underscore the complexity of plant–pollinator interactions. Further research into traits like scent and nectar composition will clarify broader ecological and evolutionary implications, deepening our understanding of co-evolution. Exploring other mediating factors (e.g., nectar chemistry, scent) and environmental variations (e.g., pollinator diversity levels) could further elucidate their adaptive significance.

## 4. Materials and Methods

### 4.1. Study Species and Locations

**Figure 9:**
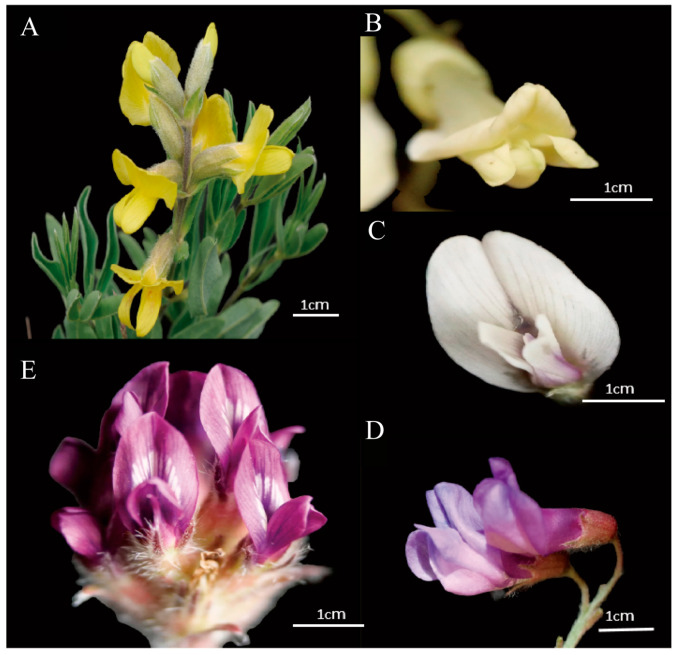
Five Papilionoideae flower displays. (**A**) *T. lanceolata*. (**B**) *S. flavescens*. (**C**) *A. galactitess*. (**D**) *V. Bungei*. (**E**) *O. ochrantha* var. *longisepala*.

The present study focuses on five species within the Papilionoideae of Fabaceae (Figure 9), which have overlapping flowering periods and are sympatrically distributed. The species under scrutiny are *Thermopsis lanceolata* R. Br., *Astragalus galactites* Pall., *Vicia bungei* Ohwi, *Oxytropis ochrantha* var. *longisepala* X. Zhao et K. Sun, and *Sophora flavescens* Alt. Sampling was carried out in June and July 2024 in the vicinity of Mapo Township, Xinglong Mountain, Yuzhong County, Lanzhou City, Gansu Province, China (35.7779424° N, 104.0149987° E).

### 4.2. Floral Morphometry and Color Measurement

Five 10 × 10 m quadrats (one quadrat per species) were systematically established in representative habitats for each plant for floral morphometric measurements. Thirty healthy plants (free from pests, diseases, and mechanical damage) were selected diagonally along the sampling plots using the systematic sampling method from 9 a.m. to 5 p.m. on a sunny, windless day during the bloom period of the natural population of each plant (defined as the 10th to 15th day of bloom of a single plant, when the stigmas were fertile, and pollen viability was >90%). One standard flower in full bloom (petals fully expanded, anthers not shed) was selected from each plant, totaling 30 flowers/species.

**Figure 10:**
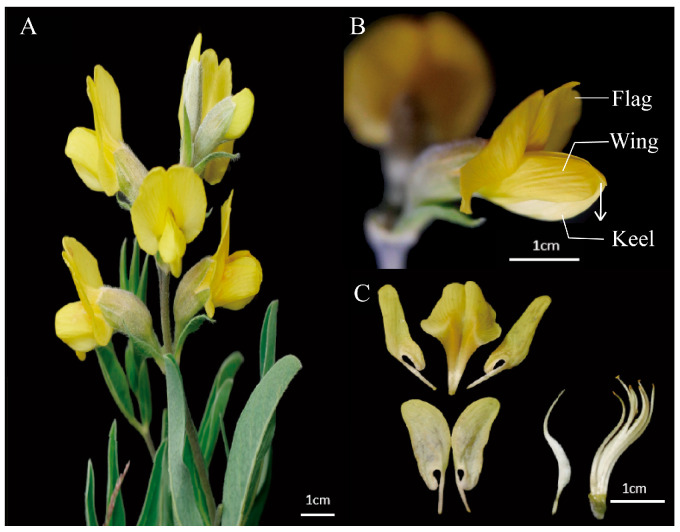
Schematic illustration of a typical Papilionoideae flower (e.g., *T. lanceolata*). (**A**) Inflorescence characteristics. (**B**) Floral structure. (**C**) Anatomical structure.

Morphological measurement index system: morphological measurements followed the KEW Plant Morphological Protocol [42] using digital vernier calipers (Guanglu 111–101, 0–150 mm, 0.01 mm, ±0.03 mm). The following parameters were measured: Flower display length: vertical measurement from the apex of the flag petal (uppermost standard petal) to the point of attachment at the base of the keel petal. Flower display width: measured perpendicular to the direction of the flower axis, the maximum horizontal span of the two sides of the wing petals after the expansion of the petals (Figure 10).

Sepal length: select the most fully developed sepal on the dorsal side, from the basal insertion point to the tip (excluding hairy appendages). Sepal width: measured perpendicular to the long axis at the widest point of the sepal. Nectar spur length: measured along a naturally curved path from where the receptacle joins the nectar spur to the end of the spur (Figure 10).

Flower color measurement: flower color was quantified using a Konica Minolta CR-400 chroma meter (Konica Minolta Sensing, Inc., Tokyo, Japan) under standardized conditions (D65 illuminant, 10° observer angle). For each flower, five replicate measurements were taken from the adaxial surface of the largest petal (central region, avoiding veins) to minimize variability. When the color of the petals was uneven, the part of the petals that had the most vibrant colors was selected for measurement. Reflectance data were converted to CIELAB color space values (L*, a*, b*, *C*, *h*), and RGB values were derived from the device’s built-in software. Measurements were taken between 09:00 and 11:00 h to ensure consistent natural lighting, and each species was represented by at least 15 individual flowers (n = 15–20 per species). The parameter L* (brightness) indicates the brightness of the color, which usually ranges from 0 (black) to 100 (white). The larger the value of L*, the brighter the color; the smaller the value, the darker the color. The parameter a* (red-green component) indicates the red-green variation of the color. When the value of a* is positive, the color is reddish; when the value is negative, the color is greenish. The parameter b* (yellow-blue component) indicates the yellow and blue components of a color. When the value of b* is positive, the color is yellow-biased; when the value is negative, the color is blue-biased. The parameter *C* (chromaticity) indicates the saturation of the color, reflecting the vividness of the color. The larger the value of *C*, the more vivid the color; the smaller the value of C, the grayer the color. The parameter *h* (hue) indicates the type of the color, that is, the angular value of the color. The parameter R (red) indicates the intensity of the red channel: the larger the value of R, the redder the color. The parameter G (green) indicates the intensity of the green channel: the larger the value of G, the greener the color. The parameter B (blue) indicates the intensity of the blue channel: the larger the value of B, the bluer the color.

To investigate the relationships between floral morphological traits and biomechanical properties, key floral characteristics were measured across five Papilionoideae species, and their correlations with flower operative strength were analyzed. Descriptive statistics (mean ± SD) were calculated in Excel. Correlations between floral traits and operative strength were analyzed using Pearson’s coefficients in SPSS 26.0 [43]. Heatmaps were visualized with R 4.3.1 (corrplot package 0.95).

### 4.3. Measurement of the Flowers Operative Strength

**Figure 11:**
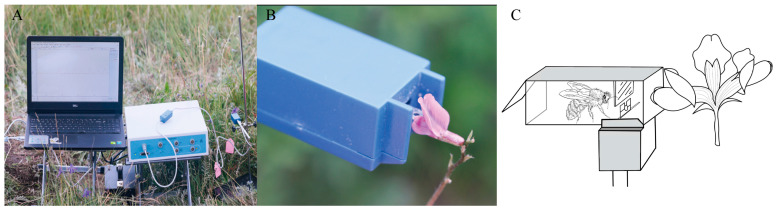
Display of experimental equipment. (**A**) Photograph of the field equipment used during the study, showing the laptop, data acquisition system, and measurement devices. (**B**) Measuring the mechanical strength required to press open keel petals during flowering using a biotensile sensor. (**C**) Device for determining the mechanical strength of pollinating insects. We constructed a culvert that allowed insects to pass through. One end of the passage could be sealed, and a transparent baffle and a metal plate with a biotension sensor were placed at the port at the other end. Flowers were placed outside the port to lure the insects. The insect had to push against the metal plate as it tries to leave, and the force exerted is continuously recorded. The maximum force exerted by the insect during this process was considered its mechanical strength.

Thirty flowers in full bloom were selected from each Papilionoideae species to determine the “flower opening strength”. The selection criteria required that the flowers—the identical specimens used to measure floral morphological characters—were structurally intact and had no obvious herbivorous damage or developmental abnormalities. We used the FT-102 biological tension sensor (BL-420s Biofunctional Experimental System and FT-102 Biotensile Sensor, 0.001–5 g, Taiman Software V1.0, Chengdu Techman Software Co., Ltd., Chengdu, China) to measure (Figure 11A,B). The operational mechanical strength of Papilionoideae flowers was determined by manually applying force to the keel petals using a metal plate attached to the sensor until the stamens and pistils became exposed (Figure 11C). Before measurements, in situ behavioral observations of flower-visiting insects were conducted to determine the natural petal-opening angle, which was simulated during manual measurements. Measurements were taken without detaching or altering the growth state of the flowers to exclude the effect of flower picking on mechanical strength. Operative strength thresholds (mean ± SD) were calculated in Excel. Interspecific comparisons used one-way ANOVA with Tukey’s HSD in SPSS 26.0 [43].

### 4.4. Observations of Flower-Visiting Insects and Analysis of Visiting Preferences

We observed and captured flower-visiting insects of these five Papilionoideae species during the peak flowering period. Ten 5 × 5 m quadrats were systematically established across representative habitats (2 quadrats/species, minimum 10 m apart) to observe. The number and behavior of all flower-visiting insects were observed and recorded from 9:00 to 18:00 each day under sunny weather conditions for a total observation time of more than 180 h [44]. If regular flower-visiting insects were found, they were captured and euthanized in vials containing ethyl acetate at the end of the flower visit. Individuals of insects in the study area (including visitors and non-visitors) were captured, and specimens were deposited with their specimen numbers in a specimen box for identification by entomologists. Two expert entomologists used a stereomicroscope to measure the anatomical traits of the insects for species identification. Relative abundance and diversity indices (Shannon’s H) were calculated in PAST 4.03. Pollinator-plant associations were tested with chi-squared tests in R 4.3.1 (stats package 4.3.1) [45].

To explore the influence of floral traits on flower-visiting insects of five Papilionoideae plants, we conducted RDA analysis on floral display (flower size and flower color) and floral rewards (nectar volume and sugar concentration) in relation to the number of flower visits by insects. Considering that there are only five plant species and four groups of floral traits, we aggregated the observed flower-visiting insects at the genus level for easier analysis. For floral traits, flower size was measured using digital calipers to record flower length, as it is a fundamental morphological characteristic that may determine whether an insect can effectively interact with the flower. Nectar volume was quantified with a micropipette, as it represents a direct floral reward. Flower nectar sugar concentration (sugar) was determined using a handheld refractometer, which measures the sugar content indicative of nutritional value. Color was selected as it is a key visual cue for insects to locate flowers. Together, these factors—color (visual attraction), flower size (morphological interaction), nectar volume, and sugar (floral rewards)—cover multiple dimensions of floral-insect interactions. Prior to RDA, all variables were tested for multicollinearity (VIF < 10) to ensure statistical reliability, and non-normally distributed data (e.g., the number of flower visits) were log10(x + 1)-transformed. The RDA was constrained by species identity to account for interspecific differences, and the significance of each explanatory variable was evaluated via Monte Carlo permutation tests (999 permutations, *p* < 0.05), ensuring rigorous statistical inference about the relationships between floral traits and the number of pollinator flower visits.

To investigate the interaction patterns between insects and plants, and to understand insect preferences for flower colors, we began by observing plant species near our study site. Through field observations and collections, we identified the flower-visiting insects associated with these plants. To analyze these interaction patterns, we mapped the relationships between the identified insect species and plant species. This involved documenting each insect species’ occurrence on each plant species and visualizing these connections using a circular network diagram. In this diagram, the thickness and color of the connections represented the strength and nature of the interactions, respectively, providing a comprehensive overview of insect–plant interactions. Subsequently, for those insect species observed to visit both Papilionoideae and non-Papilionoideae plants, we categorized all plant species into four flower-color categories through visual assessment. We then examined the flower-visiting preferences of these insect species for each color category. By tracking the pattern of each insect species’ visits to differently colored flowers, we constructed a circular diagram. In this diagram, the connections from each insect species to the color categories visually represented their preferences, with thicker connections indicating a stronger preference for specific flower colors.

### 4.5. Measurement of the Mass and Strength of Pollinators

Pollinating insects were collected at the same location as the study plants and maintained in a controlled environment with water and sucrose to measure their mass and strength [28]. Force was measured using a fabricated insect force-measuring device, and the maximum force exerted by each insect during the process was defined as its mechanical strength (Figure 10C). Flowers were positioned outside the port to lure the insects. The insect, attempting to exit, had to push against the metal plate, and the force exerted was continuously recorded. The maximum force exerted by the insect during this process was considered its mechanical strength. Linear regression (insect weight vs. strength) and *t*-tests (visitors vs. non-visitors) were performed in SPSS 26.0 [43]. Data were log10(x + 1)-transformed for normality.

## 5. Conclusions

This study provides valuable insights into the interplay between flower color and mechanical screening mechanisms in shaping plant–pollinator interactions. Specifically, the findings demonstrate that although flower color and mechanical strength do not exhibit a significant relationship, they function synergistically to filter and attract effective pollinators. The mechanical strength of flowers acts as a primary threshold, first determining which pollinators can physically access floral resources (e.g., by displacing keel petals to expose reproductive organs). In parallel, flower color serves as another cue, refining pollinator selection by attracting specific taxonomic groups—even among insects capable of overcoming the mechanical barrier. These results underscore the complexity of plant–pollinator coevolution, revealing adaptive strategies wherein plants integrate multiple traits to optimize pollination efficiency. Moreover, they carry practical implications for conserving Papilionoideae-dominated ecosystems: maintaining habitats that preserve both floral color diversity and appropriate mechanical traits could enhance pollinator specificity, thereby boosting plant reproductive success and ecosystem resilience. Looking forward, future research might explore how these findings extend to other plant families or floral trait combinations—for example, investigating whether scent or nectar properties interact with color and mechanical strength to further optimize pollination syndromes.

## Figures and Tables

**Table 1:** Flower characteristics of five Papilionoideae species (mean ± SD).

Species	Flower Length/mm	Flower Width/mm	Nectar Spur/mm	Sepal Length/mm	Sepal Width/mm	Plant Height/mm	Operative Strength/mN
*Vicia bungei*	16.46 ± 1.07 ^a^	15.62 ± 1.37 ^a^	7.41 ± 0.36 ^a^	13.17 ± 1.40 ^a^	2.87 ± 0.36 ^a^	158.2 ± 29.6 ^d^	20.25 ± 2.88 ^a^
*Thermopsis lanceolata*	13.98 ± 1.15 ^b^	9.10 ± 1.04 ^b^	7.17 ± 0.60 ^a^	10.95 ± 1.08 ^b^	1.47 ± 1.59 ^b^	264.11 ± 35.66 ^c^	18.62 ± 1.87 ^b^
*Sophora flavescens*	11.72 ± 1.59 ^d^	5.57 ± 0.74 ^e^	5.87 ± 0.79 ^c^	5.62 ± 0.88 ^e^	0.82 ± 0.76 ^c^	409.89 ± 34.34 ^a^	12.59 ± 2.23 ^d^
*Astragalus galactites*	12.51 ± 1.47 ^c^	8.01 ± 0.95 ^c^	6.79 ± 0.87 ^b^	9.48 ± 0.96 ^c^	1.06 ± 0.26 ^c^	373.66 ± 66.27 ^b^	15.92 ± 3.43 ^c^
*Oxytropis ochrantha* var. *longisepala*	12.24 ± 1.20 ^c^	6.46 ± 0.88 ^d^	6.59 ± 1.07 ^b^	7.00 ± 0.67 ^d^	0.95 ± 0.24 ^c^	167.21 ± 18.97 ^d^	13.23 ± 3.10 ^d^

Superscript letters indicate significant differences among species (N = 40, Tukey’s HSD test, *p* < 0.05).

## Data Availability

All data generated or analyzed during this study are included in this published article.

## Supplementary Materials

The following supporting information can be downloaded at: https://www.mdpi.com/article/10.3390/plants14111568/s1, Table S1: Flower-visiting insects for all plants in the sample plot; Table S2: Species numbering for plant-pollinator interaction network diagrams; Table S3: The RDA results of insects visiting flowers and plant traits; Table S4: The table of coefficients for each variable in different axes of RDA analysis; Table S5: Plant species with four flower colors after RGB colourimetric analysis.
