# Supplementary material for: Synergistic Effects of Flower Color and Mechanical Barriers on Pollinator Selection Within the Papilionoideae of Fabaceae
Source: Plants (Basel). 2025 May 22;14(11):1568. doi: 10.3390/plants14111568 (PMC12157013; doi:10.3390/plants14111568)
Supplement: Supplementary file 1 [file plants-14-01568-s001.zip › plants-3606564-supplementary.pdf]

plants

MDPI

Supplementary materials

Table S1. Flower-visiting insects for all plants in the sample plot

ORDER	FAMILY	GENUS	SPECIES	WHETHER VISITING PAPILIONOIDEAE
**DIPTERA**	Syrphidae	*Eristalis*	*Eristalis tenax*	No
**HYMENOPTERA**	Ichneumonidae	*Ichneumon*	*Ichneumon* sp.	No
	Megachilidae	*Megachile*	*Megachile sculpturalis*	Yes
			Megachile rotundata	No
			Megachile lagopoda	Yes
			Megachile manchuriana	Yes
	Apidae	*Anthophora*	*Anthophora aenieventris*	Yes
			Anthophora agama	Yes
			Anthophora fulvitarsis	Yes
			Anthophora finitima	Yes
		Apis	*Apis cerana*	No
			Apis mellifera	Yes
		Bombus	*Bombus impetuosus*	Yes
			Bombus bicoloratus	Yes
			Bombus waltoni	Yes
			Bombus lantschouensis	Yes
			Bombus kashmirensis	Yes

Table S2. Species numbering for plant-pollinator interaction network diagrams

Plant species	Number	Plant species	Number	Insect species	Number
V. bungei	P1	*Potentilla chinensis* Ser.	P18	*M. lagopoda*	B1
T. lanceolata	P2	*Taraxacum mongolicum* Hand.-Mazz.	P19	*M. manchuriana*	B2
S. flavescens	P3	*Potentilla anserina* L.	P20	*M. sculpturalis*	B3
A. galactites	P4	*Trigonotis peduncularis* Trev.	P21	*M. rotundata*	B4
O. Ochrantha var. *longisepal*	P5	*Dracocephalum heterophyllum* Benth.	P22	*A. aenieventris*	B5
Bothriospermum kusnezowii Bge.	P6	*Potentilla glabra* Lodd.	P23	*A. agama*	B6
Anemone rivularis Buch.-Ham.	P7	*Erodium stephanianum* Willd.	P24	*A. fulvitarsis*	B7
Pedicularis alaschanica Maxim.	P8	*Linum perenne* L.	P25	*A. finitima*	B8
Geum aleppicum Jacq.	P9			*A. cerana*	B9
Ligularia przewalskii Maxim.	P10			*A. mellifera*	B10
Scutellaria baicalensis Georgi	P11			*B. kashmirensis*	B11
Berberis stenostachya Ahrendt	P12			*B. impetuosus*	B12
Iris lactea Pall.	P13			*B. bicoloratus*	B13
Cirsium lanatum Spreng.	P14			*B. waltoni*	B14
Euphorbia fischeriana Steud.	P15			*B. lantschouensis*	B15
Anaphalis lactea Maxim.	P16			*E. tenax*	B16
Thalictrum aquilegifolium Linn.	P17			*Ichneumon* sp.	B17

Table S3. The RDA results of insects visiting flowers and plant traits

	Axis1	Axis2	Axis3	Axis4
Eigenvalue	27659.780	12724.041	567.385	54.374
Proportion Explained	0.675	0.310	0.014	0.001

Cumulative Proportion	0.675	0.985	0.999	1.000

Table S4. The table of coefficients for each variable in different axes of RDA analysis

Constrain	RDA1	RDA2	RDA3	RDA4
color	0.118	0.978	-0.161	0.060
flower size	-0.899	0.253	0.349	-0.079
volume	-0.675	0.425	0.272	-0.538
sugar	0.323	0.747	0.094	-0.574

Table S5. Plant species with four flower colors after RGB colourimetric analysis

Colour number	Plant species
Col1	*V. bungei*，*O. Ochrantha* var. *longisepal*，*S. baicalensis*，*I. lactea*，*E. stephanianum*，*C. lanatum*
Col2	*S. flavescens*，*A. galactites*，*E. fischeriana*，*T. aquilegifolium*，*T. peduncularis*，*D. heterophyllum*，*P. glabra*，*A. rivularis*，*A. lactea*
Col3	*T. lanceolata*，*P. chinensis* ，*T. mongolicum*，*P. anserina*，*P. alaschanica*，*G. aleppicum*，*L. przewalskii*，*B. stenostachya*
Col4	*L. perenne*，*B. kusnezowii*
